# Clinical prognostic impact of C-NLR in heart failure patients with different ejection fractions: a retrospective study

**DOI:** 10.1186/s12872-024-03714-4

**Published:** 2024-01-17

**Authors:** Fazhi Yang, Lihua Zhang, Wei Huang, Dajin Liu, Yunhong Yang, Wenyi Gu, Tao Shi, Sirui Yang, Lixing Chen

**Affiliations:** 1 Department of Cardiology, Kunming Medical University First Affilliated Hospital, City, Kunming, Yunnan Province China; 2 Department of General Medicine, Kunming Medical University First Affilliated Hospital, City, Kunming, Yunnan Province China; 3 Department of Geriatrics, The Third People’s Hospital of Yunnan Province, City, Kunming, Yunnan Province China; 4 Medical Records and Statistics Department, Kunming Medical University First Affilliated Hospital, City, Kunming, Yunnan Province China

**Keywords:** Heart failure, Inflammation, Immunity, Prognosis, Ejection fraction

## Abstract

**Objection:**

Inflammatory conditions and immune disorders may worsen the prognosis of chronic heart failure (CHF) patients. The aim of this study was to evaluate the prognostic value of a new indicator, C-NLR, composed of C-reactive protein (CRP) and neutrophil-to-lymphocyte ratio (NLR), for the risk of all-cause mortality in HF patients with different ejection fractions.

**Methods:**

A total of 1221 CHF patients admitted to the First Affiliated Hospital of Kunming Medical University from January 2017 to October 2021 were enrolled in this study. All patients were divided into 2 groups according to the median C-NLR. Kaplan–Meier survival curves were used to compare the all-cause mortality among CHF patients with different ejection fractions. Cox proportional hazards analysis was used to evaluate the relationships between variables and mortality. The predictive value of the C-NLR was assessed by using receiver operating characteristic (ROC) analyses.

**Results:**

We collected data from 1192 patients with CHF. Kaplan–Meier survival analysis revealed that patients with low LCR levels had better overall survival (OS). After multivariate adjustment Cox proportional hazards analysis, the level of C-NLR was still independently related to mortality.

**Conclusions:**

C-NLR was a competent independent predictor in HF with different ejection fractions, and routine measurement of C-NLR would help clinical doctors identify patients with a poor prognosis.

## Introduction

Due to its high morbidity and mortality worldwide, heart failure has been considered an emerging epidemic affecting approximately 38 million patients worldwide. According to the Yang 2021 study, the prevalence of heart failure (HF) continues to rise in China [1]. Both the prevalence and incidence of HF increased significantly with increasing age. The prevalence of HF was 1.38% in those aged 35 years and above, 3.09% in those aged 60 to 79 years, and 7.55% in those aged 80 years and above. The incidence of heart failure ranges from 60 to 79, reaching 720/100000 person-years among people aged years old and 1655/100000 person-years among people aged 80 years and above [1]. Chronic heart failure (CHF) is a group of complex and progressive clinical syndromes, and acute exacerbation of CHF refers to the aggravation of heart failure symptoms and/or signs in chronic heart failure patients after a stable period of time. Acute exacerbation of chronic heart failure is closely associated with death in patients. The high mortality and readmission rates of heart failure patients bring about an enormous public health burden on the country, and it is crucial to explore clinically feasible markers to help in the early identification and treatment of patients with worsening heart failure.

HF patients are divided into three categories based on left ventricular ejection fraction (LVEF) according to the 2021 European Society of Cardiology Heart Failure Guidelines, namely, HF with reduced EF (HFrEF), defined as LVEF ≤40%; HF with mildly reduced EF (HFmrEF), defined as LVEF between 41 and 49%; and HF with preserved ejection fraction (HFpEF), defined as LVEF ≥50% [2].

Currently, CRP and blood cell count-based markers are increasingly important in predicting outcomes in HF patients with ejection fractions. First, inflammation plays a critical role in the occurrence and development of cardiac hypertrophy, and heart failure has recently been recognized in several studies [3, 4]. Some inflammatory markers, such as neutrophils [5] and C-reactive protein (CRP) [6]^,^ have been confirmed to be closely related to the development of HF. In addition, lymphocytes [7], which can reflect the immune status of HF patients, are also related to the prognosis of acute exacerbation of chronic heart failure. We wanted to explore a clinically feasible joint indicator to identify high-risk HF patients, predict their prognosis and improve their survival rate.

Recently, a new index, C-NLR, which consists of both CRP and neutrophil-to-lymphocyte ratio (NLR), has been proposed, and its prognostic significance was initially validated in patients with pancreatic cancer after pancreatic resection [8]. Similar to cancer, chronic heart failure is also a systemic disease. Due to the characteristics of increased inflammation levels and decreased immune function in the process of HF, we inferred that the C-NLR might be an efficient prognostic index in worsening HF patients with different ejection fractions. However, there is no relevant research on C-NLR in HF. The aim of our study was to evaluate the clinical prognostic impact of the C-NLR in HF patients with different ejection fractions.

## Methods

### Study population

A total of 1221 medical records of patients diagnosed with acute exacerbation of chronic heart failure at the First Affiliated Hospital of Kunming Medical University from January 2017 to October 2021 were obtained and retrospectively analysed. We included HF patients who were admitted as New York Heart Association (NYHA) functional class III or IV, as well as patients with a brain natriuretic peptide (BNP) level of ≥500 pg/mL. Finally, 1192 patients remained and were included in this study, excluding those who lacked data on left ventricular ejection fraction, neutrophils, lymphocytes, or CRP,had a combination of other serious diseases (e.g., malignancies, blood diseases, or severe renal or liver dysfunction) or had no follow-up data.

### Data collection

Demographic and clinical information and electrocardiograms (ECGs) were collected on admission. Demographics, including age and sex, and clinical data consisted of NYHA cardiac functional classification, body mass index (BMI), aetiologies and treatment. Red blood cell (RBC) count, white blood cell (WBC) count, absolute neutrophil count, absolute lymphocyte count, haemoglobin (HB) level, platelet count, CRP level, myoglobin, D-dimer, troponin I and creatine kinase-MB (CK-MB) were measured directly. After 12 hours of fasting, other blood samples were collected in strict accordance with the standard procedures and sent to the laboratory of the First Affiliated Hospital of Kunming Medical University for immediate testing. Detection indicators included sodium, potassium, albumin, alanine aminotransferase (ALT), aspartate aminotransferase (AST), serum total bilirubin (STB), and conjugated bilirubin (CB). Echocardiography was completed within 3 days after admission.

Survival data were collected through telephone interviews with the patients or their families, and all-cause mortality was defined as the study endpoint.

### Calculation of NLR and C-NLR

Blood and biochemical parameters were measured for each patient after 12 hours of fasting. The NLR was calculated by dividing the neutrophil count by the lymphocyte count, and the C-NLR was calculated by the serum CRP level (mg/l) × NLR.

### Statistical analysis

We divided the patients into a low C-NLR group (C-NLR ≤ 27.56, *n* = 596) and a high C-NLR group (C-NLR > 27.56, n = 596) according to the median C-NLR. Continuous data are presented as the mean ± standard deviation (SD) when they were normally distributed; otherwise, they are presented as the median (interquartile range). Categorical data are presented as counts and percentages. For comparisons between the patient groups with different C-NLR levels, an independent sample t test was used for comparison of normally distributed data. The Mann–Whitney U test was used for comparison of nonnormally distributed data. The chi-square test was used for comparison of categorical data. In addition, to further investigate the clinical prognostic impact of C-NLR in heart failure with different ejection fraction types, all patients were divided into 2 groups based on left ventricular ejection fraction (LVEF) during statistical analysis: the HFrEF+HFmrEF group (LVEF < 50%, *n* = 728) and the HFpEF group (LVEF ≥50%, *n* = 464).

The correlation coefficient was calculated for statistical correlations between continuous variables based on Spearman’s nonparametric test. In addition, we performed stratification analysis to confirm whether the effect of CRP and NLR differed in each of the subgroups. To evaluate the relationship between C-NLR and overall survival, survival curves were plotted according to the Kaplan–Meier method and compared using the log-rank test.

Unadjusted univariate Cox proportional hazard regression analyses were applied to roughly show the impact of each variable on all-cause mortality. Variables that were significant (*p* < 0.05) in the univariate analysis were evaluated with multivariate analysis using a stepwise logistic regression model to determine independent predictors of all-cause mortality, while CRP and NLR were excluded due to direct correlation with C-NLR. The first unadjusted group was regarded as the reference group. The results are expressed as hazard ratios (HRs) with 95% confidence intervals (CIs). Four adjusted models were used for multivariate analysis. The confounders selected were significant (*p* < 0.05) in the univariate analysis or were considered influential for mortality in our model. Time-dependent receiver operating characteristic (ROC) curves and the corresponding area under the curve (AUC) were calculated to compare the predictive ability of C-NLR in HF patients with different ejections. A *p* value < 0.05 was considered statistically significant. Analyses were performed with the statistical package SPSS 26.0.

### Ethics

The study protocol was approved by the Medical Ethics Committee of the First Affiliated Hospital of Kunming Medical University and was in line with the guidelines of the World Medical Association Declaration of Helsinki. All patients gave written informed consent for their data to be electronically stored and used for research. The ethics number of the study was (2022) Ethics L No.173.

## Results

### Patient characteristics

After excluding lost visits and patients with missing data, 1192 HF patients were included in the study, including 596 in the low C-NLR level group and 596 in the high C-NLR level group. In this study, the mean age of the population was 66.82 years, and 62.1% of the patients were male. Compared with patients in the low C-NLR level group, patients in the high C-NLR level group were older and had a higher heart rate (HR), a higher proportion of NYHA IV patients, a higher prevalence of coronary disease and diabetes, and higher WBC, NEU, LYM, ALT, AST, STB, CB, creatinine, urea nitrogen, BNP, lgBNP, CRP and NLR levels (*p* < 0.05) but lower DBP, RBC, HB, sodium, albumin (Alb), TC, and eGFR levels, as well as low left ventricular end diastolic volume (LVEDV) and left ventricular end systolic volume (LVESV) (*p* < 0.05) (Table 1).

**Table 1:** Baseline characteristics according to C-NLR level

Variables	Total study Population *n* = 1192	Low C-NLR *n* = 596	High C-NLR *n* = 596	*p* value
Basic characteristics				
Sex, male, %	740(62.1)	358(60.1)	382(64.1)	0.152
Age, years	66.82 ± 12.52	64.97 ± 12.47	68.68 ± 12.31	< 0.001
BMI, kg/m^2^	23.01 ± 3.80	23.03 ± 3.91	23.00 ± 3.70	0.882
SBP, mmHg	122.05 ± 22.96	123.19 ± 22.79	120.91 ± 23.08	0.086
DBP, mmHg	76.19 ± 15.05	77.09 ± 15.41	75.30 ± 14.64	0.041
HR,	85.27 ± 21.03	82.69 ± 19.73	87.85 ± 21.96	< 0.001
beat/minute				
Smoking, %	407(34.1)	191(32.0)	216(36.2)	0.127
NYHA IV, %	442(37.1)	187(31.4)	255(42.8)	< 0.001
Aetiologies				
Coronary disease, %	616(51.7)	284(47.7)	332(55.7)	0.005
Hypertension, %	658(55.2)	326(54.7)	332(55.7)	0.727
Diabetes, %	340(28.5)	151(25.3)	189(31.7)	0.015
Peripheral vascular disease, %	376(31.5)	174(29.2)	202(33.9)	0.081
Atrial fibrillation, %	405(34.0)	204(34.2)	201(33.7)	0.854
Laboratory data				
RBC, 10^12/L	4.54 ± 0.76	4.60 ± 0.72	4.48 ± 0.80	0.005
WBC, 10^9/L NEU, 10^9/L	7.96 ± 3.84 5.55 ± 3.36	6.79 ± 2.64 4.30 ± 2.01	9.12 ± 4.46 6.80 ± 3.93	< 0.001 < 0.001
LYM, 10^9/L	1.50 ± 0.76	1.72 ± 0.82	1.27 ± 0.62	< 0.001
Haemoglobin, g/L	138.04 ± 24.14	140.10 ± 22.37	135.98 ± 25.64	0.003
Sodium, mmol/L	141.00 ± 4.43	141.65 ± 3.99	140.36 ± 4.74	< 0.001
Potassium, mmol/L	3.94 ± 0.60	3.95 ± 0.56	3.94 ± 0.64	0.759
Albumin, g/l	36.69 ± 4.54	37.81 ± 4.26	35.57 ± 4.54	< 0.001
ALT, IU/L	25.05 (16.70,42.30)	24.20 (16.53,38.48)	26.75 (16.73,49.80)	0.009
AST, IU/L	28.60 (20.10,43.28)	27.20 (20.00,37.40)	30.00 (20.53,53.33)	< 0.001
STB, μmol/L	15.80 (10.40,24.60)	15.10 (10.10,23.18)	16.30 (10.73,25.70)	0.053
CB, μmol/L	7.10 (4.60,12.00)	6.70 (4.40,10.90)	7.40 (4.93,12.88)	0.002
TC, mmol/L	3.66 (3.00,4.21)	3.67 (3.12,4.27)	3.60 (2.94,4.15)	0.016
TG, mmol/L	1.12 (0.87,1.43)	1.13 (0.86,1.51)	1.10 (0.87,1.36)	0.150
Cre, μmol/L	103.50 (83.20,134.10)	98.00 (81.60,123.85)	109.30 (85.70,144.03)	< 0.001
Urea, mmol/L	7.48 (5.72,10.26)	6.93 (5.60,9.29)	8.11 (5.91,11.63)	< 0.001
UA, μmol/L	485.10 (376.48,586.00)	474.20 (374.20,569.70)	494.73 (379.60,604.38)	0.063
eGFR, ml/min	44.10 (32.29,56.69)	47.20 (35.52,59.50)	41.11 (28.30,53.70)	< 0.001
BNP, pg/ml	1405.00 (877.48,2307.50)	1240.00 (811.25,2067.50)	1570.00 (952.25,2627.50)	< 0.001
lgBNP	3.17 ± 0.28	3.13 ± 0.27	3.21 ± 0.29	< 0.001
CRP, mg/L	7.50 (3.02,21.78)	3.02 (1.59,5.44)	21.61 (12.50,47.80)	< 0.001
NLR	3.31 (2.17,5.71)	2.52 (1.78,3.55)	4.89 (3.09,8.28)	< 0.001
C-NLR	27.56 (7.88,95.15)	7.90 (3.97,15.24)	95.02 (51.16,238.38)	< 0.001
ECG parameters and Echocardiographic data				
QRS wave, ms	106.00 (94.00,128.00)	106.00 (95.00,128.00)	106.00 (93.00,128.00)	0.257
LVEF, %	43.00 (33.00,58.00)	42.00 (32.00,58.00)	44.00 (34.00,58.00)	0.156
LVEF group				
HFrEF	523(43.9)	278(46.6)	245(41.1)	
HFmrEF	205(17.2)	91(15.3)	114(19.1)	0.087
HFpEF	464(38.9)	227(38.1)	237(39.8)	
LVEDV, ml	180.00 (126.00,238.00)	186.00 (139.25,244.00)	171.50 (118.00,230.75)	< 0.001
LVESV, ml	105.00 (55.00,158.00)	111.50 (61.00,164.00)	96.00 (51.00,150.00)	0.001
Treatment				
ACEI/ARB/ARNI, %	655(54.9)	325(54.5)	330(55.4)	0.771
Beta-blocker, %	592(49.7)	312(52.3)	280(47.0)	0.064
CRT/D,%	116(9.7)	61(10.2)	55(9.2)	0.558
SGLT2i, %	240(20.1)	123(20.6)	117(19.6)	0.665
Diuretics, %	983(82.5)	486(81.5)	497(83.4)	0.402

Differences in the normally distributed continuous variables were compared using an independent sample t test, and those in the nonnormally distributed data were compared using the Mann–Whitney U rank sum test. The χ2 test was used to compare the between-group differences in the categorical variables. The *p* values are derived from comparing the HFpEF plus HFmrEF group with the HFrEF group. A p value < 0.05 was considered indicative of statistical significance

*BMI* body mass index: *SBP* systolic blood pressure: *DBP* diastolic blood pressure: *HR* heart rate: *NYHA* New York Heart Association: *RBC* red blood cell: *WBC* white blood cell: *NEU* neutrophil: *LYM* lymphocyte: *CRP* C-reactive protein: *ALT* alanine aminotransferase: *AST* aspartate aminotransferase: *STB* serum total bilirubin: *CB* conjugated bilirubin: *TC* total cholesterol: *TG* triglyceride: *Cre* creatinine: *Urea* urea nitrogen: *UA*,uric acid: *eGFR* estimated glomerular filtration rate: *BNP* brain natriuretic peptide: *CRP* C-reactive protein: *NLR* neutrophil-to-lymphocyte ratio: *LVEF*,left ventricular ejection fraction: *LVEDV* left ventricular end diastolic volume: *LVESV*, left ventricular end systolic volume

### Linear correlation of CRP with NLR

**Fig. 1:**
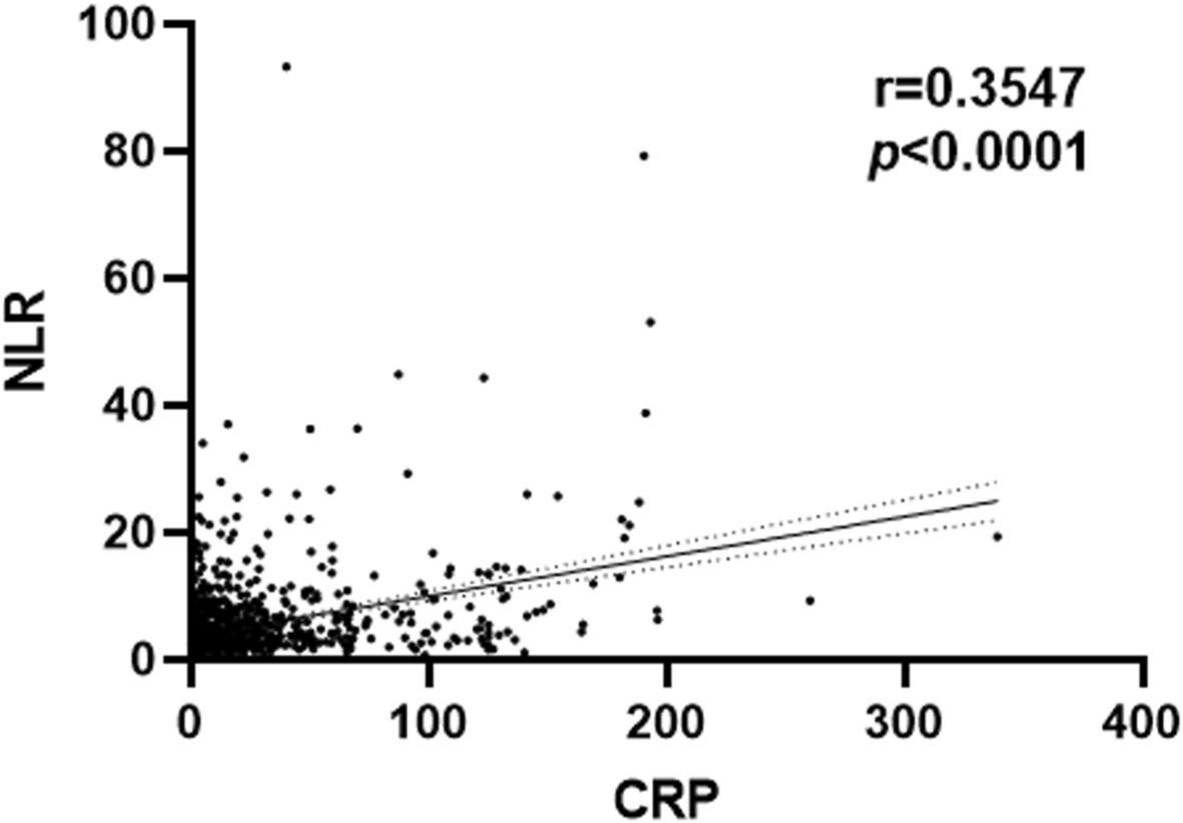
Linear correlation between C-reactive protein (CRP) and neutrophil-to-lymphocyte ratio (NLR)

We tested the correlation between CRP and NLR in patients with HF. The correlation analysis based on Spearman’s nonparametric test revealed a weak linear correlation between CRP and NLR in the total participant population [Spearman’s correlation coefficient (r): 0.44, *p* < 0.001] (Fig. 1).

### Subgroup analyses

A subgroup analysis was performed to determine the association between CRP, NLR and all-cause mortality in HF patients. In the subgroup analyses, after adjusting for age, sex, BMI, SBP, DBP, NYHA, urea nitrogen, creatinine, eGFR, serum sodium, haemoglobin, albumin, TC and lgBNP and using Group 1 (CRP ≤ 7.50 + NLR ≤ 3.31) as a reference, both Group 2 (CRP > 7.50 + NLR ≤ 3.31) and Group 4 (CRP > 7.50 + NLR > 3.31) had statistical significance and were associated with higher all-cause mortality rates (Group 2, HR: 2.121, 95% CI: 1.628–2.765, *p* < 0.001; Group 4, HR: 2.488, 95% CI: 1.936–3.197, *p* < 0.001) (Table 2).

**Table 2:** Subgroup analysis of the association between C-reactive protein (CRP) and neutrophil-to-lymphocyte ratio (NLR)

	Unadjusted		Adjusted	
	HR(95% CI)	*p* value	HR(95% CI)	*p* value
CRP				
≤7.50	Reference		Reference	
> 7.50	2.876(2.408,3.434)	< 0.001	2.226(1.850,2.679)	< 0.001
NLR				
≤3.31	Reference		Reference	
> 3.31	1.780(1.504,2.106)	< 0.001	1.256(1.050,1.503)	0.013
Combined categories				
Group 1:CRP ≤ 7.50 + NLR ≤ 3.31	Reference		Reference	
Group 2:CRP > 7.50 + NLR ≤ 3.31	2.451(1.887,3.184)	< 0.001	2.121(1.628,2.765)	< 0.001
Group 3:CRP ≤ 7.50 + NLR > 3.31	1.358(1.013,1.820)	0.041	1.100(0.817,1.482)	0.529
Group 4:CRP > 7.50 + NLR > 3.31	3.956(3.133,4.996)	< 0.001	2.488(1.936,3.197)	< 0.001

Adjusted for age, sex, BMI, SBP, DBP, NYHA, urea nitrogen, creatinine, eGFR, serum sodium, haemoglobin, albumin, TC and lgBNP

### Survival analyses based on C-NLR

**Fig. 2:**
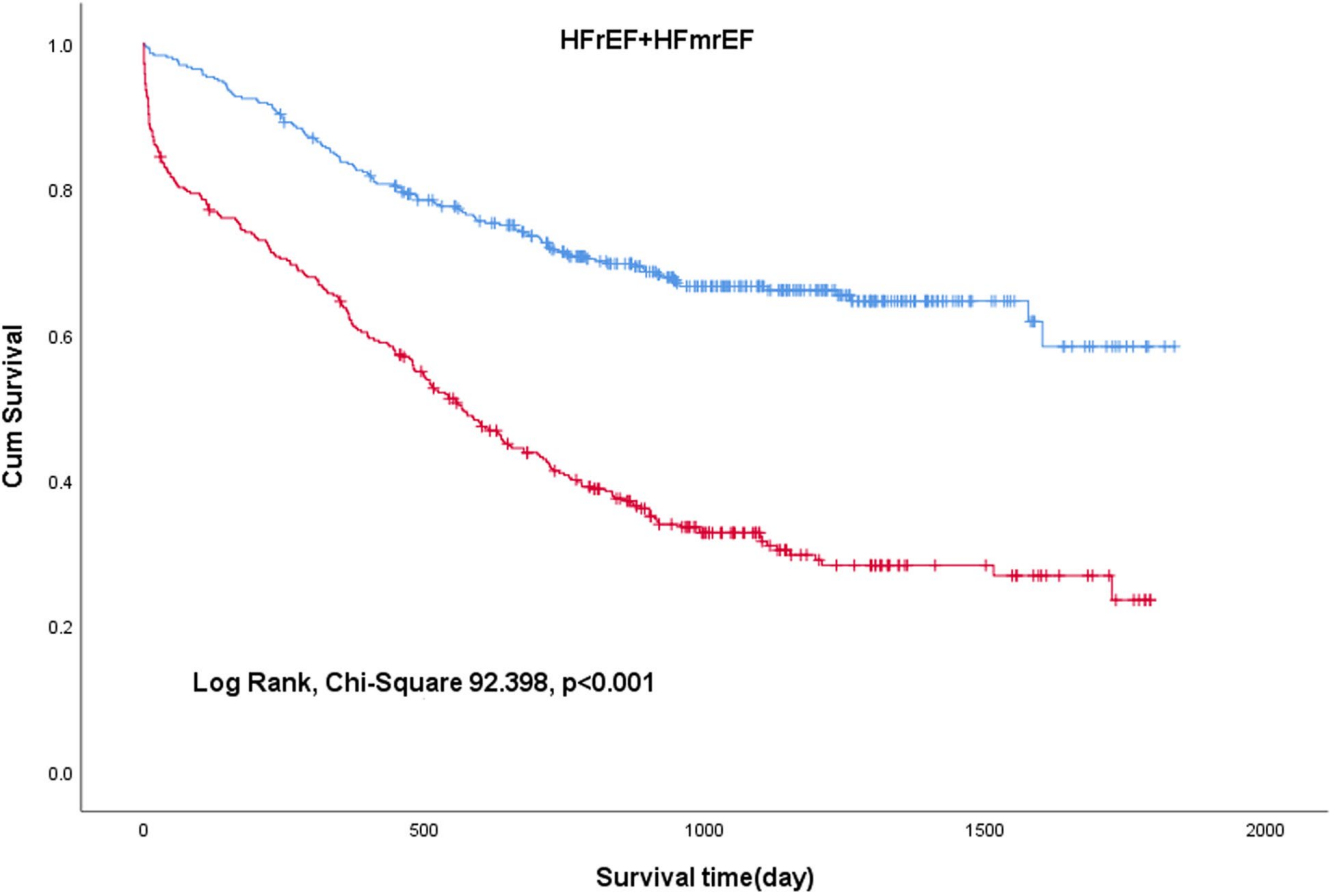
Kaplan–Meier curves for all-cause mortality in patients with HFrEF+HFmrEF

**Fig. 3:**
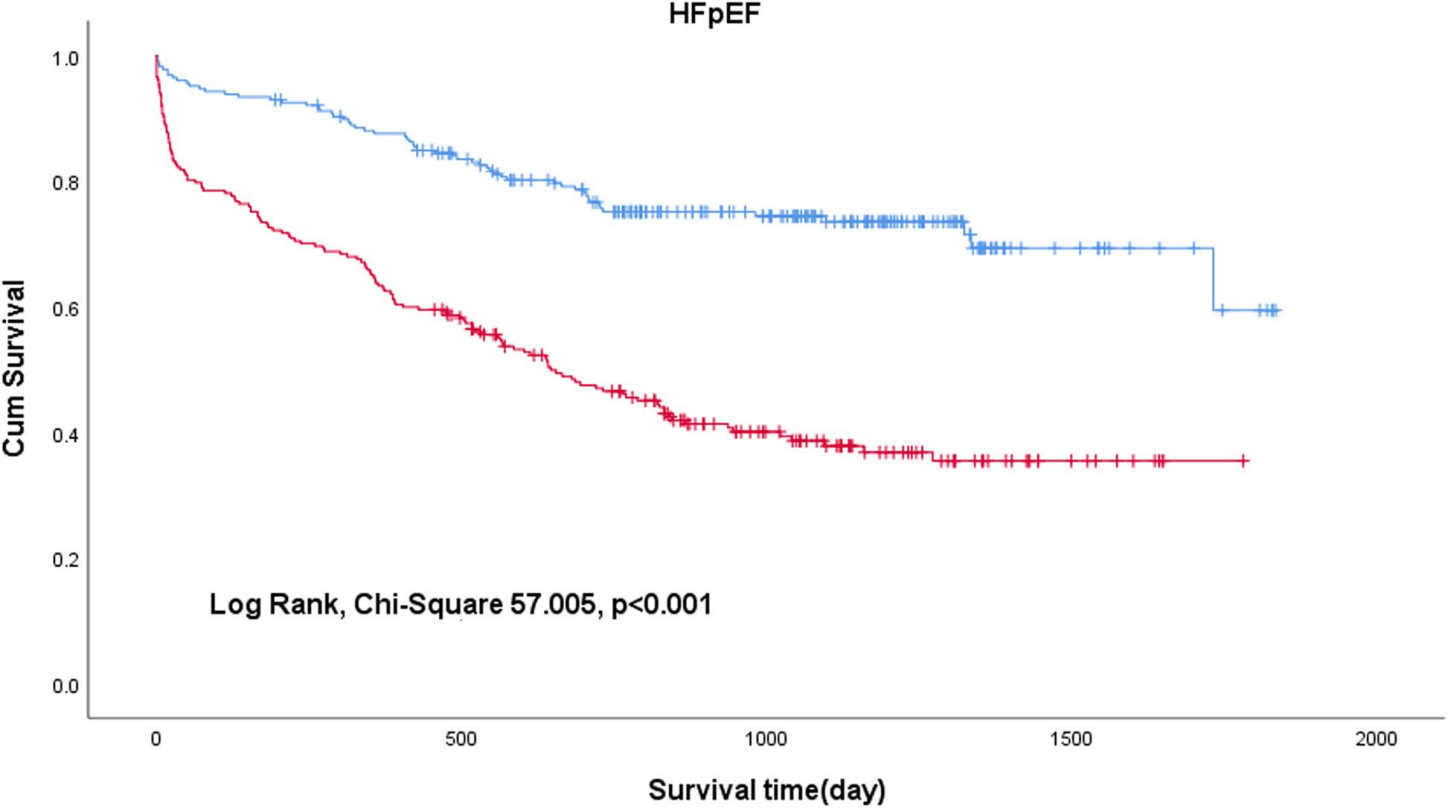
Kaplan–Meier curves for all-cause mortality in patients with HFpEF

To investigate the effect of the C-NLR on the prognosis of patients with HF, unadjusted Kaplan–Meier analysis was implemented. The Kaplan–Meier survival analysis demonstrated that low levels of C-NLR (≤27.56) were significantly associated with better overall survival (OS) in HFrEF+HFmrEF (Fig. 2) (log-rank test, chi-square 92.398, *p* < 0.001) and HFpEF (Fig. 3) (log-rank test, chi-square 57.005, *p* < 0.001).

### C-NLR as an independent predictor of all-cause mortality

**Fig. 4:**
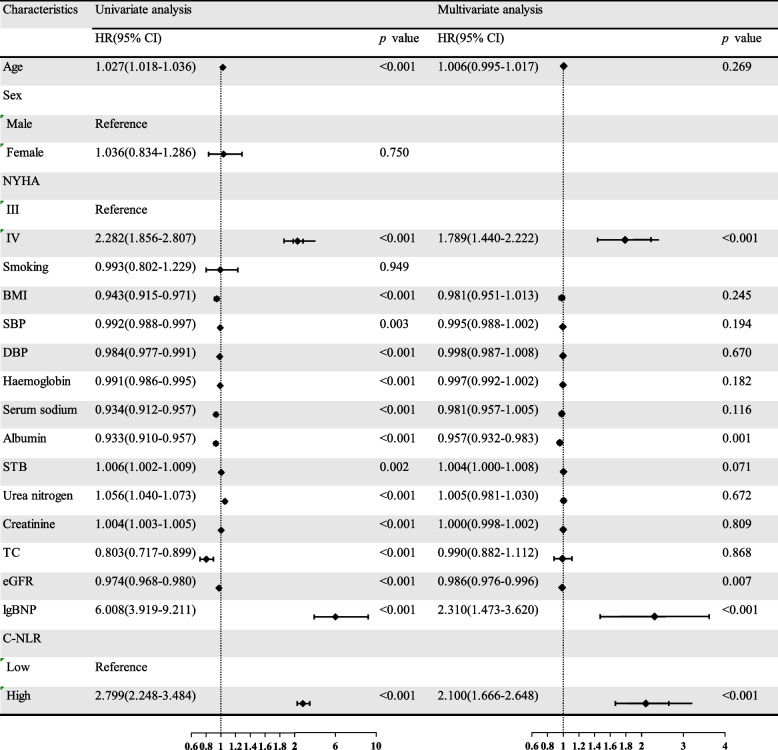
Univariate and multivariate analyses of clinicopathological variables in relation to all-cause mortality in HFrEF+HFmrEF

**Fig. 5:**
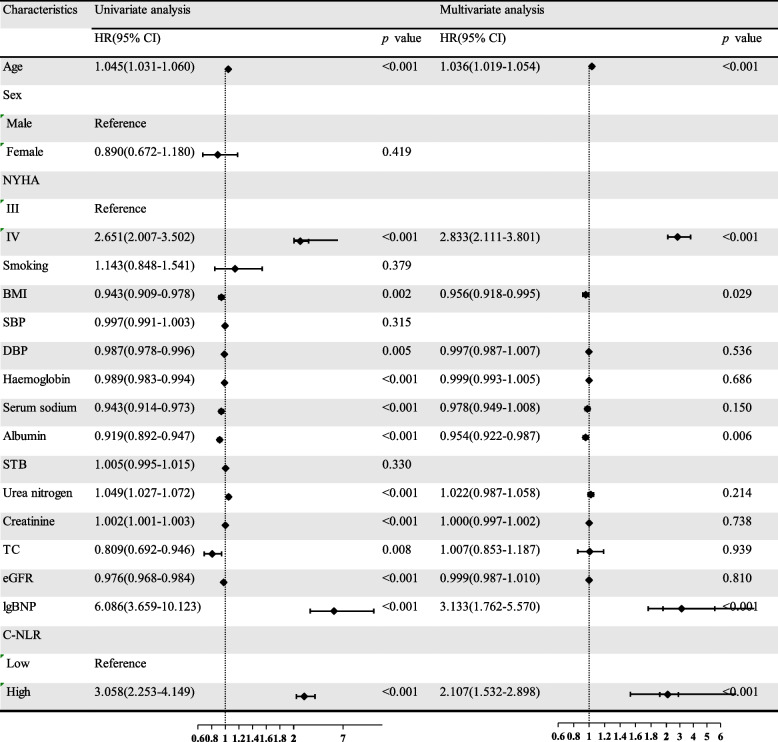
Univariate and multivariate analyses of clinicopathological variables in relation to all-cause mortality in HFpEF

For the HFrEF+HFmrEF group, in the multivariate analysis, NYHA IV (HR: 1.789, 95% CI: 1.440–2.222, *p* < 0.001), albumin (HR: 0.957, 95% CI: 0.932–0.983, *p* = 0.001), eGFR (HR: 0.986, 95% CI: 0.976–0.996, *p* = 0.007), lgBNP (HR: 2.310, 95% CI: 1.473–3.620, *p* < 0.001), and C-NLR (HR: 2.100, 95% CI: 1.666–2.648, *p* < 0.001) were the independent and significant predictors of overall survival (Fig. 4). For the HFpEF group, in the multivariate analysis, age (HR: 1.036, 95% CI: 1.019–1.054, *p* < 0.001), NYHA IV (HR: 2.833, 95% CI: 2.111–3.801, *p* < 0.001), BMI (HR: 0.956, 95% CI: 0.918–0.995, *p* = 0.029), albumin (HR: 0.954, 95% CI: 0.922–0.987, *p* = 0.006), lgBNP (HR: 3.133, 95% CI: 1.762–5.570, *p* < 0.001), and C-NLR (HR: 2.107, 95% CI: 1.532–2.898, *p* < 0.001) were the independent and significant predictors of overall survival (Fig. 5).

Four adjusted models were used for the multivariate analysis. In Model 1, the adjusted covariates were age and sex. In Model 2, the adjusted covariates were age, sex, body mass index (BMI), systolic blood pressure (SBP) and diastolic blood pressure (DBP). In Model 3, the adjusted covariates were age, sex, BMI, SBP, DBP, NYHA, urea nitrogen, creatinine and estimated glomerular filtration rate (eGFR). In Model 4, the adjusted covariates were age, sex, BMI, SBP, DBP, NYHA, urea nitrogen, creatinine, eGFR, serum sodium, haemoglobin, albumin, total cholesterol (TC) and lgBNP. In both the HFrEF+HFmrEF group and HFpEF, Cox proportional hazards analysis for age, sex, BMI, SBP, DBP, NYHA, urea nitrogen, creatinine, eGFR, serum sodium, haemoglobin, albumin, TC and lgBNP, high LCR levels (reference: low LCR levels) (HFrEF+HFmrEF, HR: 2.146, 95% CI: 1.704–2.703, *p* < 0.001; HFpEF, HR: 2.031, 95% CI: 1.471–2.804, *p* < 0.001) were independently related to mortality after multivariate adjustment (Table 3).

**Table 3:** Cox proportional hazards models for the association of C-NLR and all-cause mortality

Model	HFrEF+HFmrEF		HFpEF	
	HR(95% CI)	*p* value	HR(95% CI)	*p* value
Unadjusted	2.799(2.248–3.484)	< 0.001	3.058(2.253–4.149)	< 0.001
Model 1	2.659(2.133–3.314)	< 0.001	2.692(1.978–3.662)	< 0.001
Model 2	2.687(2.154–3.352)	< 0.001	2.698(1.979–3.677)	< 0.001
Model 3	2.482(1.984–3.105)	< 0.001	2.372(1.731–3.252)	< 0.001
Model 4	2.146(1.704–2.703)	< 0.001	2.031(1.471–2.804)	< 0.001

*HR* hazard ratio: *CI* confidence interval

Reference: low C-NLR

Model 1: Adjusted for age and sex

Model 2: Adjusted for the variables included in Model 1 and BMI, SBP, DBP

Model 3: Adjusted for the variables included in Model 2 and NYHA, urea nitrogen, creatinine, eGFR

Model 4: Adjusted for the variables included in Model 3 and serum sodium, haemoglobin, albumin, TC, and lgBNP

### Predictive ability of C-NLR in HF patients with different ejection fractions

**Fig. 6:**
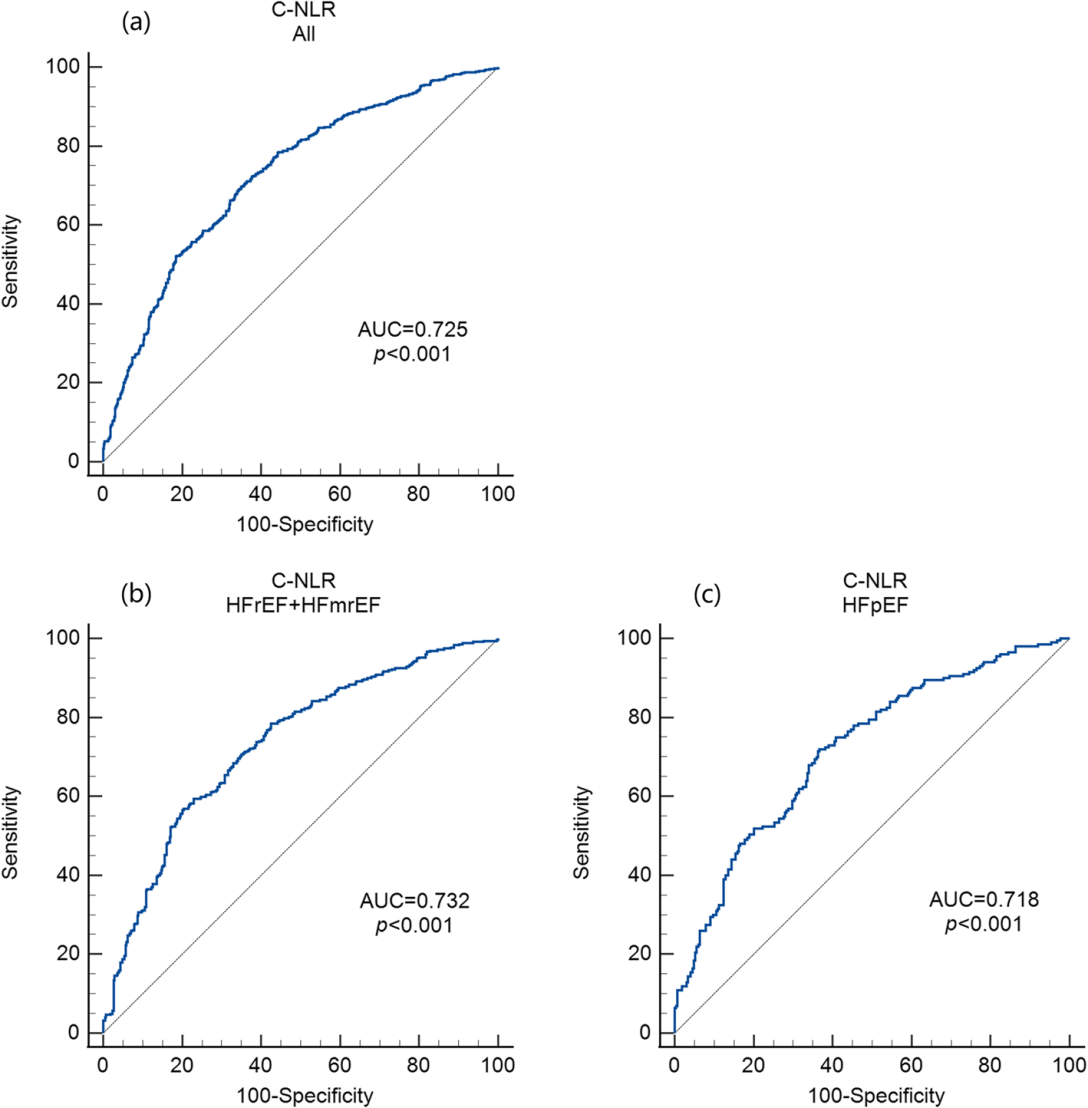
Time-dependent receiver operating characteristic (ROC) curves of C-NLR with the reference line for all-cause mortality in all HF patients (**a**), HFrEF+HFmrEF patients (**b**) and HFpEF patients (**c**)

To investigate the predictive value of C-NLR for all-cause mortality in HF patients with different ejection fractions, we generated ROC curves. In the ROC curves, the C-NLR had an AUC of 0.725 (95% CI: 0.699–0.751) (Fig. 6a). The optimal cut-off point was 26.421, and the sensitivity and specificity were 0.690 and 0.657, respectively. For the HFrEF+HFmrEF group, the AUC of C-NLR was 0.732 (95% CI: 0.698–0.764) (Fig. 6b). For the HFpEF group, the AUC of the C-NLR was 0.718 (95% CI: 0.675–0.759) (Fig. 6c).

## Discussion

In this retrospective study, a novel predictive index, CNLR, was introduced that combines CRP and NLR. This study revealed a weak linear correlation between CRP and NLR and suggested that CRP-based and blood cell count-based systemic inflammatory responses play an important role in predicting CHF aggravation. To the best of our knowledge, this is the first study reporting the prognostic value of the C-NLR in HF patients with different ejection fractions.

In the Kaplan–Meier analysis, high C-NLR was shown to be significantly associated with short survival times (log rank test, *p* < 0.001), preliminarily suggesting that C-NLR is a worthwhile metric to study. In the univariate Cox proportional hazards analysis, OS was significantly worse in patients with a higher C-NLR (*p* < 0.001). In the multivariate adjustment Cox proportional hazards analysis, high C-NLR levels were independently associated with a high risk of all-cause mortality. For HFrEF+HFmrEF, the risk of mortality in patients with high C-NLR levels was 2.100-fold higher than that in patients with low C-NLR levels (*p* < 0.001). For HFpEF, the risk of mortality in patients with high C-NLR levels was 2.107-fold higher than that in patients with low C-NLR levels (*p* < 0.001). After four adjusted models were used for the multivariate analysis, C-NLR was still independently related to mortality (*p* < 0.001). According to the ROC curves, the AUC of C-NLR was 0.732 in HFrEF+HFmrEF and 0.718 in HFpEF. Therefore, we have reason to consider that the C-NLR has a great predictive ability for all-cause mortality in HF patients with different ejection fractions. The possible mechanisms are described as follows.

First, CRP has been traditionally used as a marker of infection and cardiovascular events, and its elevation may reflect an aggravation of the inflammatory response in patients with HF [9]. Increased CRP levels were associated with a 2.8-fold increased risk of developing HF in Framingham’s study [10]. CRP is an acute inflammatory protein and is mainly synthesized by interleukin-6 (IL-6)-dependent hepatic biosynthesis, and elevated CRP levels in the serum are an independent predictor of cardiovascular disease. CRP levels have been confirmed to be associated with prognosis in patients with heart failure, atherosclerotic disease, myocarditis and atrial fibrillation, and CRP itself is toxic to the myocardium [11, 12]. CRP, a classic acute marker of inflammation, is deposited at sites of inflammation and tissue damage. Furthermore, there is evidence to suggest that CRP is not only a marker of inflammation but also plays a positive role in the inflammatory process. CRP can lead to the release of pro-apoptotic cytokines and inflammatory mediators, including interleukin-1β (IL-1β), tumour necrosis factor-α (TNF-α), and reactive oxygen species, by activating complement and binding to the Fc receptor of IgG [13]. Patients with acute exacerbation of chronic heart failure exhibit activation of pro-inflammatory cytokines, possibly through the activation of the renin angiotensin aldosterone system and sympathetic nervous system [14]. In addition, CRP can induce the apoptosis of human coronary vascular smooth muscle cells and accelerate the cardiac remodelling process [15]. CRP has also been shown to play a role in mediating low-density lipoprotein uptake in macrophages, which is implicated in atherogenesis, and studies have also shown that CRP is an independent predictor of CHF risk after stroke or transient ischaemic attack (TIA) [14, 16].

Second, neutrophils, which are also an inflammation indicator, are the first responders to infection and injury and the first cells that arrive at the site of damage. In addition to haemodynamic and neurohormonal disorders, it is increasingly recognized that inflammation plays a critical role in the occurrence and development of cardiac hypertrophy and heart failure. The inflammatory response can affect the development and prognosis of HF through various pathways, such as cardiomyocyte apoptosis and fibrosis, further leading to a decline in myocardial function [17, 18]. Tang’s study showed that neutrophils play an essential role in the pathogenesis and progression of HF and that chronic angiotensin II infusion activates the neutrophil KLF2/NETosis/thrombosis pathway, further leading to myocardial hypoxia, cell death and hypertrophy. In addition, a study has shown that the beneficial effects of ACE inhibitors in heart failure may be partially due to anti-inflammatory and neutropenia effects [19]. Meanwhile, neutrophils can also lead to immunosuppression by inducing apoptosis of lymphocytes [20]. Du Clos’s study showed a correlation between the localization of CRP in neutrophil infiltrates [21]. Our research showed a linear correlation between CRP and NLR, consistent with Du Clos’s research.

Third, lymphocytes play an important role in the immune process, and the a lymphocyte count may reflect the severity of neurohormonal and immune system disturbances [22]. Research has shown that relative lymphocytopenia is an independent predictor of mortality in HF patients and may help us identify elderly CHF patients and stratify their risk [23]. The decrease in the relative percentage of lymphocyte count in HF patients has been considered a marker of physiological stress response, associated with increased release of endogenous catecholamines or cortisol [24]. Studies have shown that potential mechanisms for the reduction in lymphocyte count in HF patients may include the activation of the hypothalamus-hypophysis-adrenal axis, the result of changes in behavioural patterns such as malnutrition and smoking and the increased serum cortisol blood levels [23]. The activation of the neuroendocrine and inflammatory systems may be the cause of the decreased lymphocyte count in heart failure patients and may also lead to immune system dysregulation in some CHF patients, contributing to heart failure progression [7, 25, 26].

The NLR is a prognostic marker for hospitalization and mortality in patients with HF. The NLR, which is calculated from the neutrophil count and lymphocyte count, reflects the balance between the innate (i.e., neutrophils) and adaptive (i.e., lymphocytes) immune responses in the body. Studies have shown that the mechanism of increased NLR in patients with heart failure may be an increase in neutrophils by systemic inflammation or stimulation of the release of granulocytic myeloid-derived suppressor cells in the bone marrow. These cells have immunomodulatory effects and are able to suppress lymphocyte responses [27]. Currently, a novel combination indicator, C-NLR, consisting of CRP and NLR, has been proposed and confirmed to be related to the poor prognosis of pancreatic cancer patients [8]. In addition, Wang’s study showed that the C-NLR is also a reliable predictor of the long-term prognosis of bladder cancer patients after radical cystectomy [28]. Similar to cancer, HF is a systemic disease that activates inflammatory responses. Several studies have revealed that CRP and NLR are independent prognostic factors in HF [27, 29]. Therefore, we believe that the C-NLR could also be used to assess the host systemic inflammatory response and immune status and the prognosis in HF patients. The advantage of these markers is that they are readily available and do not require specialized equipment for the measurements. In our study, low levels of C-NLR (≤27.56) were significantly associated with better OS, and C-NLR was a competent independent predictor in HF patients with different ejection fractions. HF patients with low levels of C-NLR (i.e., low levels of CRP and/or NLR) have a better prognosis, indicating that inflammatory conditions and immune disorders may worsen the prognosis of HF patients. There has been no report on the prognostic efficacy of the C-NLR in HF patients thus far, and we demonstrated for the first time the relationship between the C-NLR and the prognosis of acute exacerbation in CHF patients with different ejection fractions.

As a result, it is reasonable to speculate that C-NLR is a reliable prognostic indicator for HF patients with different ejection fractions. Nonetheless, there are still potential limitations, such as our research being a retrospective study, and uncontrolled confounding variables cannot be excluded. We mainly investigated patients with an NYHA class III or IV for enrolment, and these results may not be applicable to populations with less severe HF symptoms. Therefore, a larger scale of research is needed in the future.

## Conclusions

The C-NLR could be a competent independent predictor in HF patients with different ejection fractions. High C-NLR levels were significantly associated with a higher risk of all-cause mortality. Therefore, routine measurement of the C-NLR would help clinical doctors identify patients with a poor prognosis of acute exacerbation of chronic heart failure and optimize individual therapy, ultimately improving their prognosis.

## Data Availability

No datasets were generated or analysed during the current study.
